# Matching Ecosystem Services Supply and Demand through Land Use Optimization: A Study of the Guangdong-Hong Kong-Macao Megacity

**DOI:** 10.3390/ijerph18052324

**Published:** 2021-02-26

**Authors:** Wenjing Wang, Tong Wu, Yuanzheng Li, Hua Zheng, Zhiyun Ouyang

**Affiliations:** 1 State Key Laboratory of Urban and Regional Ecology, Research Center for Eco-Environmental Sciences, Chinese Academy of Sciences, Beijing 100085, China; wjwang_st@rcees.ac.cn (W.W.); tongwu15@outlook.com (T.W.); zhenghua@rcees.ac.cn (H.Z.); 2 College of Resources and Environment, University of Chinese Academy of Sciences, Beijing 100049, China; 3 School of Resources and Environment, Henan University of Economics and Law, Zhengzhou 450046, China; yzli_pili@outlook.com

**Keywords:** ecosystem services, land use allocation, urban sustainability, Guangdong-Hong Kong-Macao, China

## Abstract

Shortfalls and mismatches between the supply and demand of ecosystem services (ES) can be detrimental to human wellbeing. Studies focused on these problems have increased in recent decades, but few have applied land use optimization to reduce such spatial mismatches. This study developed a methodology to identify ES mismatches and then use these mismatches as objectives for land use optimization. The methodology was applied to the Guangdong-Hong Kong-Macao “Greater Bay Area” (GBA), a megacity of over 70 million people and one of the world’s largest urban agglomerations. Considering the demand for a healthy and secure living environment among city-dwellers, we focused on three ES: heat mitigation, flood mitigation, and recreational services. The results showed large spatial heterogeneity in supply and demand for these three ES. However, compared to current conditions in the GBA, our model showed that optimized land use allocation could better match the supply and demand for heat mitigation (number of beneficiaries increased by 15%), flood mitigation (amount of population exposed to flood damage decreased by 37%), and recreation (number of beneficiaries increased by 14%). By integrating land use allocation and spatial mismatch analysis, this methodology provides a feasible way to align ES supply and demand to advance urban and regional sustainability.

## 1. Introduction

Today, over 50% of the world’s population lives in urban areas—a figure that will increase to 68% by 2050. Rapid urban expansion and human activities have significant impacts on the natural environment, exacerbating the air pollution, the heat island effect, and flood risks, among other problems [1]. Managing urban land use to meet the demand of human wellbeing is a pressing challenge for sustainable development.

Ecosystems provide a range of direct and indirect services to reduce risks and enhance human wellbeing, now widely known as “ecosystem services” [2]. ES are the benefits people obtain from ecosystems [3,4], and they form links between environmental systems and human society [5,6]. An ES framework is a strong tool for improving decision-making efficacy in the allocation of limited natural resources [7,8].

Understanding the complex spatial relationships between ecosystem services supply and demand (ESSD) can help inform environmental management and generate useful information for integrating ES frameworks into decision-making processes [9,10]. Furthermore, integrating ES knowledge—i.e., information about ES suppliers and sink locations—in land use planning is instrumental to making polices that reducing ESSD mismatches [11,12]. For instance, tools such as i-tree landscape (https://landscape.itreetools.org/, accessed on 21 February 2021) allow the prioritization of tree planting based on several supply and demand indicators. Additionally, the quantification of ES supply and demand could help estimate the impacts of land use policy or planning, limiting the negative impacts of proposed plans on ES supply and maximizing positive impacts by meeting ES demand [13].

Mapping ESSD mismatches can reveal areas of ES supply shortfall and provide land managers with relevant insights for spatial planning [14,15]. ESSD mismatches have been used to identify potential areas that may be targeted for ecological restoration [16]. For example, based on the supply of and demand for flood regulation, areas that have a high potential to mitigate downstream flood risk through land use modifications have been identified [17]. Some authors have maintained that assessing multiple ESSD mismatches can help to better design urban green infrastructure where it is needed [8,18], and some have attempted to propose the achievement of urban self-sufficiency by optimizing ESSD at the local scale [19]. Although there have been numerous studies on integrating ES mismatches into planning, the optimization of land use to reduce ESSD mismatches remains a gap in the existing research.

Increasing ES supply through land management to reduce ESSD mismatches is a common policy goal of assessment and mapping studies [15]. Protecting natural forests and grasslands [20], maintaining diverse landscapes [21], increasing urban green space [22], and transferring tree species [23] have often been practiced. Some authors have developed relevant assessment and optimization tools. Numerous optimization algorithms have been applied to optimize land use allocation, including ant colony algorithms [24], genetic algorithms [25], nondominated sorting genetic algorithm-II [26], and artificial immune algorithms [27]. For example, CoMOLA (Constrained Multi-objective Optimization of Land use Allocation) is a landscape optimization tool that finds optimal options for multiple objectives [28], and SAORES (a spatially explicit assessment and optimization tool for regional ecosystem services) is designed to integrate assessment and optimization of ES for ecological restoration and management of the Chinese Loess Plateau [29]. Nonetheless, few studies have integrated optimization into landscape management and planning to reduce or minimize ESSD mismatches.

The aim of this paper is to propose a methodology that integrates ES and land use allocation to minimize ESSD mismatches through a multi-objective optimization model. More specifically, this paper develops an approach that, based on an ESSD assessment, identifies spatial mismatches, translates them into objectives for optimization, and estimates the improvements after optimizing land use. This approach is then applied to the Guangdong-Hong Kong-Macao “Greater Bay Area” (GBA), a “megacity” of over 70 million people in southern China.

## 2. Materials and Methods

### 2.1. Study Area and Data Sources

**Figure 1:**
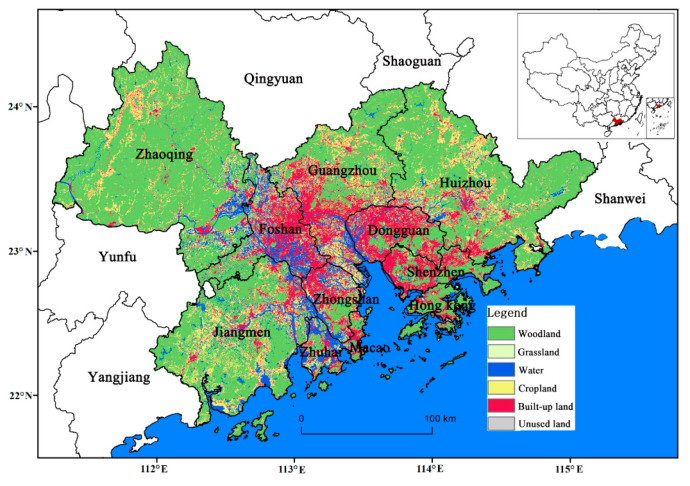
Land use and land cover (LULC) types in the “Greater Bay Area” (GBA).

The GBA is consists of 11 cities (Guangzhou, Shenzhen, Foshan, Dongguan, Zhuhai, Zhongshan, Zhaoqing, Jiangmen, Huizhou, Hong Kong, and Macao) (Figure 1). The main land use/land cover types in the GBA are forest, cropland, and urban land (Figure 1). The GBA has a southern subtropical climate with heavy rainfall during the rainy season (from April to September) and an average annual temperature of 21.4–22.4 °C; it experiences an average annual precipitation of 1600–2300 mm. Additionally, there are 1900–2200 sunshine hours per year. The GBA covers 56,000 km^2^, had a combined population of approximately 71 million at the end of 2018, and is one of the largest and most populous urban agglomerations in the world [30]. However, because of the combined effects of the complex regional terrain and the East Asian monsoon climate, flooding and high temperatures occur frequently during the summer [31]. Therefore, meeting the needs of residents for relevant ES (e.g., flood mitigation, heat mitigation, and urban green space recreation) is an important challenge for land management in this region.

Land use and land cover (LULC) data in 2018, with a spatial resolution of 30 m, was provided by the Resources and Environmental Scientific Data Center (RESDC) of the Chinese Academy of Sciences (CAS) (http://www.resdc.cn/ accessed on 11 June 2019). Population distribution data with a 100 m resolution in 2018 was obtained from WorldPop (https://www.worldpop.org/ accessed on 26 June 2019). Landsat 8 OLI_TIRS satellite remote sensing images were downloaded from the U.S. Geological Survey (http://earthexplorer.usgs.gov/ accessed on 23 August 2019) to map the land surface temperature (LST) in the GBA. Due to severe cloud obstruction in 2018, this study used the most complete and cleanest remote sensing images in the summer daytimes (from June to September) of 2019. The LST data with spatial resolution of 100 m was estimated through a practical split-window algorithm from the Thermal Infrared Sensor (TIRS) onboard Landsat 8 [32].

### 2.2. Selecting ES and Assessing Supply and Demand

#### 2.2.1. The Selection of ES

Mismatches are not a problem for some ES that can move between regions, but others require a local balance between demand and supply [33]. Considering the situation of the study area, the stakeholders (e.g., government, enterprises, residents) were first defined [34]; based on the stakeholders and their demands for ES, the ES status (surplus or deficiency) and goals for specific stakeholders were identified and confirmed. This study then chose three locally self-sufficient ES that are of particular importance to the GBA’s metropolitan population due to their centrality to human health, security, and wellbeing. The selected ES in this study are (1) heat mitigation, (2) flood mitigation, and (3) recreational space. The following paragraphs provide a brief introduction to each ES.

1. The urban heat island effect (UHI) undermines the thermal comfort and health of city-dwellers [35]. Furthermore, it can increase the use of air conditioning, which increases energy use and worsens pollution [36], and causes heat stress-associated mortality and illness [37].
2. Because of the expansion of impervious surfaces, urban regions are extremely vulnerable to flood hazards caused by heavy precipitation [38]. Climatic change and urbanization are expected to exacerbate the impacts of changing precipitation patterns and to increase the demand for water-related ES in the future [39].
3. Access to green spaces for recreation can enhance the wellbeing of city-dwellers, especially in dense urban areas [40]. Recreation space can also decrease mortality and morbidity [41], provide stress-alleviating experiences, contribute to emotional health [42], and strengthen community relationships [43].

#### 2.2.2. Assessment of ES Supply and Demand

ES supply can be assessed using biophysical models (e.g., the Soil Conservation Service curve number method), participatory methods (e.g., questionnaires, expert elicitation), monetary valuation (e.g., the market prices and cost avoidance method), ecological footprints, and other metrics [9,33]. ES demand can be assessed using social criteria (e.g., environmental quality standards, policy goals, targets for urban green space provision), participatory methods, models, and other metrics [44,45].

(1) Heat mitigation

Supply: Heat mitigation supply (*S_ce_*) is mainly provided by vegetation and water bodies [46]. The heat mitigation supply of vegetation is provided through several ways, including shading, heat reflectance, and evapotranspiration. Shading and heat reflectance can reduce the heat storage, while evapotranspiration can increase the latent heat exchange to mitigate the heat island effect [47,48]. Water bodies mainly reduce the temperature through the evapotranspiration process. Green and blue spaces not only provide local heat reduction, but can also effectively cause neighborhood cooling due to increased air movement and heat exchange [49]. In our analysis, grassland was ignored, since it covered only a small proportion of the study area and therefore had little capacity for daytime heat mitigation [50]. To calculate the local direct heat mitigation (°C), heat mitigation distance (m), and indirect benefits (°C) to neighboring areas, we randomly chose 400 forest patches and 400 water body patches on the LST map and generated buffers that were equally sliced into 30 annuluses with 30 m intervals. Finally, we calculated the average LST within each annulus, and then obtained the three abovementioned heat mitigation parameters for forest and water space.

Demand: Heat mitigation demand (*D_ce_*) was defined as the UHI intensity [51]. The UHI intensity was calculated by using an urban–rural comparison [52]. If the LST of a given pixel was lower than that of the rural background area which was defined as cropland [52], *D_ce_* was zero; otherwise, *D_ce_* was calculated as follows:

(1) $$D_{ce}=T_{i}-\frac{1}{n}\sum_{j=1}^{n}Tb_{j},$$

where *T_i_* is pixel i’s LST (℃), n is the total number of pixels within the rural background area, and $Tb_{j}$ is pixel j’s LST (℃) within the rural area.

(2) Flood mitigation 

Supply: Flood mitigation supply ($S_{fm}$) is an ecosystem’s capacity to mitigate floods by intercepting, absorbing, or detaining storm water from heavy rainfall [17]. The difference between precipitation and runoff was defined as the supply. In this study, the Soil Conservation Service curve number method [53] was used to estimate rainfall runoff and flood mitigation supply. *S_fm_* was calculated as follows:

(2) $$S_{fm}=\left(P-Q\right)\times A,$$

where $S_{fm}$ is the flood mitigation supply (m^3^); P is precipitation with duration of one hour and a return period of 50 years based on rainfall monitoring data of each city in the GBA (m); Q is the runoff (m) estimate based on the SCS-CN (Soil Conservation Service Curve Number) model as shown in Equation (3); and A is the pixel area (m^2^). Furthermore, Q was determined by

(3) $$Q=\{\begin{array}{c} \frac{{\left(P-I_{a}\right)}^{2}}{P+0.8S}\;\;\;\;P\geq 0.2S\\ \;\;\;\;\;\;\;\;\;0\;\;\;\;\;\;\;\;\;\;\;\;\;\;\;P<0.2S \end{array},$$

where $I_{a}$(mm) is the initial abstraction of the rainfall ($I_{a}=0.2S$) and S is potential maximum retention or infiltration (m), which was calculated as

(4) $$S=\frac{25,400}{CN}-254,$$

where CN is a dimensionless parameter ranging from 0 to 100. The CN values were derived from previous studies, mainly based on land cover categories and soil types [54,55].

Demand: Flood mitigation demand ($D_{fm}$) was defined as the amount of population influenced by floods, and it is related to flood exposure and vulnerability [56,57]. The $D_{fm}$ was calculated as

(5) $$D_{fm}=Exposure\times Vulnerability,$$

where Exposure refers to the population at risk of flooding and Vulnerability indicates the percentage (%) of the exposed population given a certain flood depth, as determined by depth-loss curves [57]. We calculated and summed the $D_{fm}$ of 377 catchments.

(3) Recreation

Supply: Recreation supply (S_r_) has two aspects: (1) the distance at which a given green space is located from people and (2) the size of the green space [58]. We considered only the green spaces within walking distance of the population distribution areas. Based on previous studies, we defined green spaces as areas larger than 1 ha within walking distance (300 m straight-line distance, approximately 500 m path distance, approximately 10–15 min by foot) for recreation, and areas larger than 10 ha within medium walking distance (700 m straight-line distance, approximately 1000 m path distance, around 20 min by foot) for recreation [43].

Demand: Recreation demand (D_r_) is based on the green space around population distributions. Pedestrians tend to use green spaces within a 300 to 1000 m walking distance of their home [59,60]. To match the recreation supply, there should be at least 1 ha of green space in the 300 m buffer for the population distribution area, and more than 10 ha of green space in the 700 m buffer for the population distribution area. To calculate these values, population distributions and density maps are needed.

### 2.3. Assessing ES Mismatches

The spatial relationship between ES supply and demand may be balanced (the supply is equal to or greater than demand) or deficient (the supply is lower than the demand). ESSD (mis)matches were identified via statistical tools or by overlapping different thematic maps [61]. This approach requires supply and demand to be assessed at the same scale to obtain a budget or ratio that indicates an ES undersupply, neutral balance, or oversupply [45].

The mismatch analysis of ESSD was derived from the differences between the ES supply and demand (based on the cell size) to optimize land use allocation [62]. The shortfalls and mismatches of the three ES were calculated using different indicators and methods. For heat mitigation, the mismatch was defined as the heat mitigation demand (°C). The shortfall between flood mitigation supply and demand was the runoff depth (m) in each spatial unit, which was calculated using Equation (3). A scoring method was used to assess the mismatches of outdoor recreation supply and demand. The criterion for mismatches of outdoor recreation supply and demand was defined as whether there was green space within a certain distance (700 m for areas larger than 10 ha, 300 m for areas between 1 and 10 ha) from populated areas [63] (Table 1). For unpopulated areas, the mismatch of outdoor recreation supply and demand was zero.

### 2.4. Setting the Optimization Objectives and Identifying Constraints

#### 2.4.1. Description of Optimization Objectives and Constraints

If a targeted ES supply does not match the corresponding demand (the ES supply is lower than the demand), reducing the ESSD mismatch is defined as an optimization objective. Considering the different factors and objectives involved in the definition of spatial suitability, several objectives (e.g., maximizing the number of beneficiaries of ES, achieving maximum compactness of land use to avoid land fragmentation) may be developed when directly taking into account all the relative factors [28,64]. Multi-objective optimization problems can be solved either by integrating all the objectives into one single function by assigning a weight to each objective, or by generating multiple solutions simultaneously based on Pareto-based methods [65]. In this study, we defined three objectives for land use allocation: minimizing ESSD mismatches, maximizing the number of ES beneficiaries, and maximizing land use compactness; we then adopted equal weights to integrate them into one function. Increasing the ES supply to reduce ESSD mismatches was the main objective in the area where ES supply was lower than the demand in this study. Moreover, since the ES supply is significantly more effective in densely populated areas [66], maximizing the number of ES beneficiaries was another objective. In addition, since the concentration of similar land use types improves efficiencies in land resource and energy utilization [67], we also included land use compactness (which can improve efficiency in land utilizations) as an objective.

The constraints are defined based on ecological, economic, and social restrictions, as well as other indicators. Transition rules that define possible land use transformations often take constraints, as well as the permissible total areas for different land use classes, into account [68]. We proposed two constraints to optimization: the area of land use and the rules for land use transformations. We reduced ESSD mismatches by increasing the amount of green and blue space to increase the ES supply. Based on the targets for blue and green spaces in the Pearl River Delta National Forest Urban Agglomeration Construction Plan (2016–2025), the Integrated Planning of the Ecological Security System in the Pearl River Delta Region, and the Guangdong Province General Land Use Plan (among other official planning documents), constraints were defined to limit the scale of new green and blue spaces. Forestland, water bodies, and grassland were projected to increase by approximately 300 km^2^, 60 km^2^, and 40 km^2^, respectively. The rule for land use transformations was that existing built-up land and water bodies could not be converted to other land uses in the process of optimization.

#### 2.4.2. Mathematical Formulations for Land Use Allocation

The defined objectives can be expressed by the following formulas. The different ESSD mismatches were normalized to ensure their comparability.

(6) $$f_{1}=Min\overset{Row}{\underset{i=1}{\sum }}\overset{Clo}{\underset{j=1}{\sum }}ESSD_{ce}\left(i,j\right),$$

(7) $$f_{2}=Min\overset{Row}{\underset{i=1}{\sum }}\overset{Clo}{\underset{j=1}{\sum }}ESSD_{fm}\left(i,j\right),$$

(8) $$f_{3}=Min\overset{Row}{\underset{i=1}{\sum }}\overset{Clo}{\underset{j=1}{\sum }}ESSD_{r}\left(i,j\right),$$

(9) $$f_{5}=Max\overset{K}{\underset{k=1}{\sum }}Comp_{k},$$

where $ESSD_{ce}\left(i,j\right)$ is the normalized supply and demand match for heat mitigation supply and demand in cell (*i*, *j*); $ESSD_{fm}\left(i,j\right)$ is the normalized supply and demand match for flood mitigation in cell (*i*, *j*);$\;ESSD_{r}\left(i,j\right)$ is the normalized supply and demand match for recreation in cell (*i*, *j*); and $\;Comp_{k}$ is the compactness of the *k*th land use.

We aimed to minimize ESSD mismatches through the allocation of new planned green and blue spaces. In an earlier study, increasing the ES supply was found to be significantly more effective in regions with high population densities [66]. Therefore, the goals for ES in this study can be achieved by converting the area with the greatest ESSD mismatches and highest population density to green and blue space in order to supply ES. Land use suitability analysis determines the extent to which a given piece of land is suitable for a specific use [69]. We chose ESSD matching and normalized population density to evaluate green and blue spaces. It was considered optimal when green and blue spaces were allocated to the areas with the greatest ESSD mismatches and highest population density. Based on an equally weighted sum of the mismatches of the target ES, we integrated multiple objectives to optimize blue and green spaces. Based on assessments of ES supply, the suitability of cell (*i*, *j*) for forest, water body, and grassland can be expressed as follows:

(10) $$Suit_{forest}\left(i,j\right)=POP\left(i,j\right)\times \left[ESSD_{ce}\left(i,j\right)+ESSD_{fm}\left(i,j\right)+ESSD_{r}\left(i,j\right)\right],$$

(11) $$Suit_{water}\left(i,j\right)=POP\left(i,j\right)\times \left[ESSD_{ce}\left(i,j\right)+ESSD_{fm}\left(i,j\right)\right],$$

(12) $$Suit_{grass}\left(i,j\right)=POP\left(i,j\right)\times \left[ESSD_{fm}\left(i,j\right)+ESSD_{r}\left(i,j\right)\right],$$

### 2.5. Optimizing Land Uses

A modified ant colony optimization (ACO) model was employed in our study; this approach simultaneously combines the suitability and compactness of land use based on a weighted sum of these two functions to search for optimal spatial configurations [70]. We used the ACO landscape optimization tool Geographical Simulation and Optimization System (http://www.geosimulation.cn accessed on 6 September 2019). ACO algorithms support user-defined target functions and weights of multiple objectives and allow for basic land use constraints.

**Figure 2:**
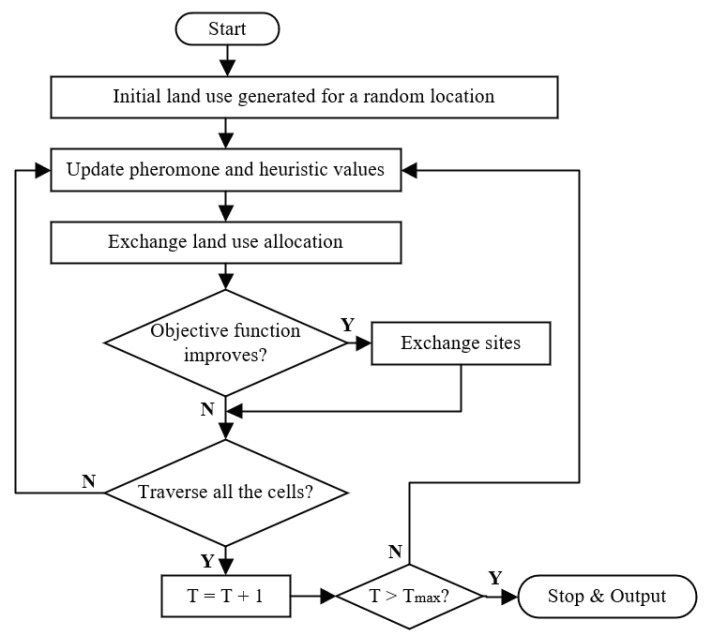
Flow chart of ant colony optimization (ACO) for land use allocation.

The flow chart of our ACO algorithm for land use optimization is shown in Figure 2. Land use can be optimized by using the pheromone feedback of ants. We imported the parameters for suitability of land use, number of ants according to the target area of land use, number of iterations, pheromone importance factor (α), visibility importance factor (β), pheromone persistence (ρ) to enable pheromone evaporation, and pheromone reward factor (q). These parameters (α, β, ρ, q) were determined as described in previous studies [70]. The basic process flow of the optimization module includes the following steps: (1) construct the initial land use allocation, which either is based on imported, actual land use data or is randomly generated; (2) produce new solutions by evaluating the value of the objective function; and (3) stop the execution when the algorithm converges or reaches the maximum level. After running the optimization algorithm, we obtained the optimized allocations of forests, water bodies, and grassland.

## 3. Results

### 3.1. Stakeholder Analysis and ES Assessment

The GBA is an urban agglomeration with over 70 million people, most of whom live in cities. Therefore, the stakeholders in the study area are primarily urban dwellers. The goal of this ESSD assessment was to reduce the shortfalls and mismatches between ES supply and demand through the optimal allocation of land use. We assessed ESSD and the optimized land use allocation to meet the stated goals.

Based on the green space and water body samples in the GBA, the mean observed direct urban heat mitigation was 3.78 °C for forests and 3.22 °C for water bodies. The indirect benefit was 2.44 °C within a 150 m buffer for forests and 1.92 °C within a 180 m buffer for water bodies. The green and blue spaces provided a cumulative heat mitigation service to 34.24 million people in the GBA. Nonetheless, there was still 13,900 km^2^ of land in the central part of the GBA affected by UHIs, with an average effect of 2.82 °C.

The flood mitigation service was assessed to be 3.19 × 10^9^ m^3^ for a 50-year pluvial flood. However, the estimated rainstorm-generated surface runoff was 2 × 10^9^ m^3^, affecting approximately 147,700 people in 377 watersheds.

Green space-derived recreation service was provided to 47.78 million people in the GBA, and 10 million people were within the service radius of community green spaces (1–10 ha) and large green spaces (>10 ha). However, 32% of people in the GBA were not within the service radius of a green space.

### 3.2. Levels of (Mis)Matches between ES Supply and Demand

**Figure 3:**
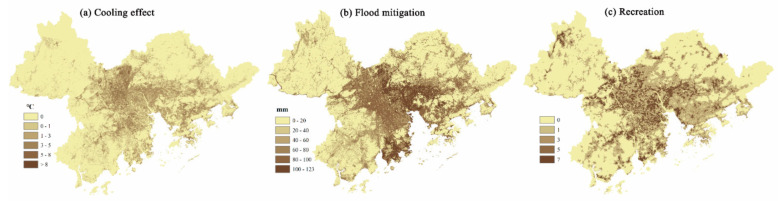
Mismatches between the supply and demand for the target ecosystem services (ES) in the GBA.

Although the mismatches for the three targeted ES were mainly distributed in the “inner city” areas of the GBA that are primarily covered with impervious surfaces, they were not spatially coincident (Figure 3). The mismatches for heat mitigation were concentrated in the middle of the GBA. Because of a heavy rainfall and high impervious surface ratio, the degree of mismatch for flood mitigation was higher in Dongguan and Zhuhai. The spatial distribution of recreation was different from that of heat mitigation and flood mitigation in that it was not scattered in areas with a lack of green space.

### 3.3. Multi-Objective Land Resource Allocation Optimization

**Figure 4:**
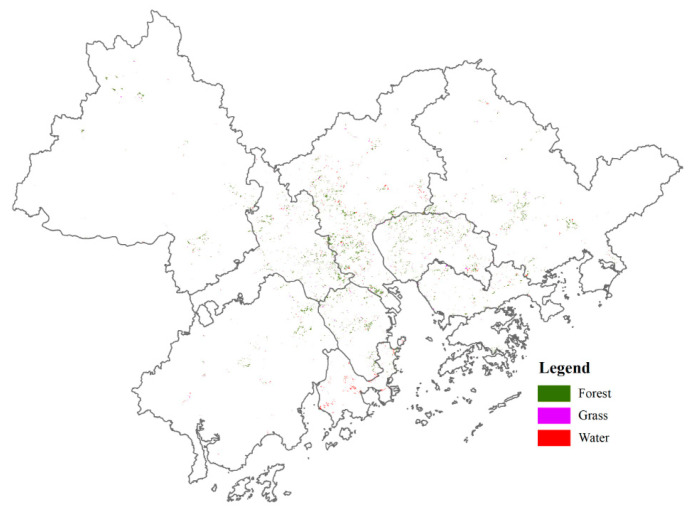
The allocation of the new green and blue spaces.

We ran an ACO model multiple times to ideally obtain for each cell a probability of land use transition. The number of iterations was set to 1000, but a close inspection revealed that after 200 iterations, most land use cells were at locations that were balanced, and the land use pattern started to stabilize after 200 iterations. The distribution of newly added green and blue spaces totaling 400 km^2^ is presented in Figure 4. Although the new green and blue spaces accounted for only 0.71% of the GBA’s total area, their optimal allocation improved ESSD matching significantly. The number of beneficiaries of heat mitigation and recreation services increased by 5.09 million and by 6.5 million, respectively, and the number of people suffering from flooding decreased by 36.73% (Table 2).

## 4. Discussion

Shortfalls and mismatches between the supply and demand of ES are common phenomena that undermine cities’ ability to cope with severe impacts such as floods and UHIs, and these impacts will only become more common as climate change continues apace [61]. One critical goal for land management is to reduce ESSD mismatches [8]. However, minimizing these mismatches by optimizing land use allocation remains difficult for a variety of technical and practical reasons [71]. Many case studies have shown that the optimization of land use allocation can achieve maximum ES supply through optimization algorithms [29,68]. However, improving wellbeing has multiple dimensions (e.g., basic material needs for health and safe living conditions) [3], and it is infeasible to allocate land use without considering residents’ demands. Therefore, our study developed a methodology for integrating the supply and demand of ES and designed an optimization module for land use allocation to minimize ESSD mismatches.

Traditional urban planning focuses on the share of maintained green spaces or permeable areas, but ignores the different ecological functions and ES provided by different types of ecological components [72]. In recent years, many urban planning policies have attempted to go beyond traditional indicators and have advocated for the inclusion of ES in decision-making [73]. Policymakers usually opt to increase the amount of green and blue spaces to maintain the balance of ecosystems and enhance the ES supply [15,22]. These plans often achieve desirable outcomes, but do not necessarily meet the overall policy goal of minimizing mismatches and shortfalls between the supply and demand of ES through land use planning [74]. Our methodology defined the minimization of ESSD mismatches and the maximization of the number of beneficiaries as objectives for land use optimization. As a result, land use optimization covering 400 km^2^ (approximately 0.71% of the GBA’s total area) provided 5.09 million (14.87% growth) additional beneficiaries with urban heat mitigation and by 6.5 million (13.61% growth) additional beneficiaries with recreation access, while at the same time reducing the number of people suffering from floods by 36.73%.

The proposed approach can be integrated into frameworks of urban land planning to identify potential intervention points for improving sustainability. Our approach can spatially quantify how much given ES need to be increased and identify where demand needs to be adjusted to achieve a regional equilibrium of ESSD. Our methodology is an innovative and potentially highly useful tool for urban planning, since it depends on urban residents’ perspectives on what is needed; it therefore increases the magnitude of the contributions to their security, health, and wellbeing from new green and blue spaces. Moreover, this study provides a way to address ES supply-demand problems by linking ES to local conditions, which can contribute to making ES operative in the context of regional land use planning. In this case study, we selected three ES based on spatial scale and the likely demands of local city-dwellers. Since the spatial area of service-providing units varies from the microscale (e.g., recreation) to the macroscale, matching ESSD can occur at many scales [75,76]. In this study, three local-scale ES (heat mitigation, flood mitigation, and recreation) were chosen to optimize land use allocation to achieve a local balance between demand and supply [33]. Moreover, these three ES are of particular importance to the GBA. The GBA has a subtropical oceanic climate and frequently experiences extreme weather events, especially storms and UHIs, during the hot summer season [77]. In addition, green space for recreation is widely recognized as being critical for the physical and mental health of dense urban populations [78]. Since the GBA is a megacity with extremely high resident density, opportunities for green space-based recreation can potentially reduce the incidence of diseases, such as obesity and cardiovascular disease [79], and increase cognitive development in children [80]. Therefore, while heat mitigation, flood mitigation, and recreation are not the only important ES for this region, they are undeniably central to the health and security of residents.

Landscape configuration can greatly affect ES supply; therefore, it was considered in this study. Landscape richness is highest when ecosystem cells are spread over as many patches as possible, while compactness is maximized when forming a single ecosystem patch [28]. Landscape richness and heterogeneity were expected to have positive effects on pollination and biodiversity, especially for species that use more than one cover type [81]. However, more experiments showed predominantly negative effects of landscape fragmentation on ES supply [82]. Fragmentation also decreases ES through interacting with other drivers such as climate change and human disturbance [83]. The degree of landscape compactness and connectivity strongly contributed to ES supply [82]. When given a fixed area of green space, the compact patches can provide a stronger cooling effect on local and surrounding areas than a fragmented distribution [84]. Additionally, increasing forest compactness positively impacts water yield and net primary productivity [85]. Addressing ESSD mismatches through land use optimization is an important approach to sustainable urbanization. Despite the innovations presented in this study, our methodology is nonetheless a first-generation effort, and as such has several limitations that require attention in future research. Since the purpose of the study is to propose a methodology to apply land use optimization to reduce ESSD mismatches, the details of assessing mismatches and objectives were simplified. For example, we assumed population density was constant and did not consider changes in population density that may occur with land use change; and we only used summer daytime LST to quantify UHI, rather than taking seasonal UHI and nighttime LST into account. Due to data limitations, we aggregated four target functions into one objective using equal weights to allocate land use, instead of assigning different weights according to the demand of different sociodemographic groups, distinguished by factors such as age, income, and education, or according to the value of different ES. For example, older people are likely to be more sensitive to high temperatures and therefore have a higher demand for heat mitigation than younger people [86], while children have a higher demand for recreational spaces [80]. Additionally, measuring the value of ecosystem services was widely applied for evaluating government policy and land use planning. Through bringing the value of ES into a single monetary metric, the value of different ES can be assigned as weights to integrate three objectives as one function. Since, the locations for maximizing different types of ES are not spatially coincident, integrating mismatches of multiple ESSDs into one objective makes sense. However, assigning weights for different ES to meet multiple demands was difficult, and needs additional work to be resolved. Given these considerations, some potentially important directions for future research include obtaining data on sociodemographic characteristics to construct models that assess varying ES demands among different groups, and assessing the monetary value of different ES.

It is also worth noting that more than 90% of the new 400 km^2^ of blue and green spaces in our study came at the expense of cultivated land, which indicates the existence of tradeoff relationships between agricultural production and the three ecosystem services in this study. Therefore, the loss of income for local households as a result of farmland conversion is a social and economic cost that is not considered in our analysis. If these households can find alternative livelihood activities or obtain payments for ES that are equal to or higher than their lost rural incomes, the practical rationale for the optimization of land use in our study would be even stronger. Thus, evaluating whether agricultural lands can be converted into ecological land to reduce ESSD mismatches, and assessing the consequent losses of income for residents, can help policymakers design policies that properly compensate the residents. 

Additionally, some uncertainties of this method need to be considered. (1) Some ecological parameters were taken from empirical studies performed in similar spatial and climatic settings, but nevertheless the parameters should be treated with care. For example, parameter $I_{a}$ in Equation (3) was proposed to use 0.2 in the GBA; however, some studies used 0.05 instead of 0.2 for urban environments. (2) This study focused on green spaces only for recreation service, which excluded water bodies where many people would spend time for recreation. Some studies showed the importance of green spaces in inner-city areas for recreation as compared to water bodies [87]. Moreover, the standard of demand for recreation, which has often been proposed by planning and research, was 300 to 1000 m walking distance for pedestrians for green spaces [88,89]. To calculate the matching of supply and demand of recreation, this study only focused on green spaces for recreation. (3) The cooling service of grasslands was not considered in this study. Because urban green space was usually composed of grasslands, forests, and shrubs, there were few patches of grassland in main urban areas in the GBA with high intensity of heat island, especially the large patches of grass. The grassland is mainly distributed in areas surrounding forests in the suburbs. Additionally, grass is less effective than tree canopy or water bodies for LST cooling [50,90]. Therefore, no significant cooling effect was found for grasslands in the GBA.

## 5. Conclusions

ESSD mismatch is a common phenomenon at specific scales. This paper provides a methodology that integrates ES and land use allocation to reconcile mismatches between ES supply and demand through multi-objective optimization. This approach was applied in the GBA, and it was found that the supply did not meet the demand for three important ES (heat mitigation, flood mitigation, and green space recreation), especially in the “inner city” areas. The mismatches for these three ES were then used as the optimization objective. After allocating 400 km^2^ (accounting for 0.71% of the GBA’s total area) of green and blue spaces, ESSD matching was greatly improved: the number of beneficiaries of the heat mitigation and recreation services increased by 5.09 million and 6.5 million, respectively, and the number of people suffering from flooding decreased by 36.73%. Our methodology extends the work of previous studies and provides a practical approach for analyzing the statuses and matching the distribution of multiple ES to further urban sustainability.

## Figures and Tables

**Table 1:** Criteria for (mis)match of outdoor recreation supply and demand.

Green Space Larger than 10 ha in 700 m Distance	Green Space Area between 1 and 10 ha in 300 m Distance	Score
√	√	1
√	×	3
×	√	5
×	×	7

**Table 2:** The changes of ES supply and demand under optimized land use allocation.

ES	Type	Original Land Use	Optimized Land Use	Change (%)
Heat mitigation	Area (km^2^)	48,116	48,821	1.47
	Beneficiaries (millions)	34.23	39.32	14.87
Flood mitigation	Runoff (10^9^ m^3^)	31.97	32.16	0.59
	Population suffering flooding (people)	147,723	93,464	−36.73
Recreation	Area (km^2^)	48,142	49,617	3.06
	Beneficiaries (millions)	47.78	54.28	13.61
